# Preoperative Imaging with [^18^F]-Fluorocholine PET/CT in Primary Hyperparathyroidism

**DOI:** 10.3390/jcm11102944

**Published:** 2022-05-23

**Authors:** Franziska J. Dekorsy, Leonie Beyer, Christine Spitzweg, Ralf Schmidmaier, Andrei Todica, Arnold Trupka, Clemens C. Cyran, Frank Berger, Roland Ladurner, Petra Zimmermann, Thomas Knösel, Peter Bartenstein, Christian Lottspeich, Vera Wenter

**Affiliations:** 1 Department of Nuclear Medicine, University Hospital, LMU Munich, 81377 Munich, Germany; leonie.beyer@med.uni-muenchen.de (L.B.); andrei.todica@med.uni-muenchen.de (A.T.); peter.bartenstein@med.uni-muenchen.de (P.B.); vera.wenter@med.uni-muenchen.de (V.W.); 2 Department of Internal Medicine IV, University Hospital, LMU Munich, 81377 Munich, Germany; christine.spitzweg@med.uni-muenchen.de (C.S.); ralf.schmidmaier@med.uni-muenchen.de (R.S.); christian.lottspeich@med.uni-muenchen.de (C.L.); 3 Department of Endocrine Surgery, Starnberg Hospital, 82319 Starnberg, Germany; arnold.trupka@klinikum-starnberg.de; 4 Department of Radiology, University Hospital, LMU Munich, 81377 Munich, Germany; clemens.cyran@med.uni-muenchen.de (C.C.C.); frank.berger@med.uni-muenchen.de (F.B.); 5 Department of Surgery, University Hospital, LMU Munich, 81377 Munich, Germany; roland.ladurner@martha-maria.de (R.L.); petra.zimmermann@med.uni-muenchen.de (P.Z.); 6 Department of Surgery, Martha-Maria Krankenhaus, 81377 Munich, Germany; 7 Institute of Pathology, Faculty of Medicine, University Hospital, LMU Munich, 81377 Munich, Germany; thomas.knoesel@med.uni-muenchen.de

**Keywords:** fluorocholine PET/CT, parathyroid adenoma, noninvasive imaging

## Abstract

Primary hyperparathyroidism (pHPT) is a common endocrine disorder due to hyperfunctioning parathyroid glands. To date, the only curing therapy is surgical removal of the dysfunctional gland, making correct detection and localization crucial in order to perform a minimally invasive parathyroidectomy. ^18^F-Fluorocholine positron emission tomography/computed tomography (^18^F-FCH PET/CT) has shown promising results for the detection of pHPT, suggesting superiority over conventional imaging with ultrasounds or scintigraphy. A total of 33 patients with pHPT who had negative or equivocal findings in conventional imaging received ^18^F-FCH PET/CT preoperatively and were retrospectively included. A pathological hyperfunctional parathyroid gland was diagnosed in 24 cases (positive PET, 72.7%), 4 cases showed equivocal choline uptake (equivocal PET, 12.1%), and in 5 cases, no enhanced choline uptake was evident (negative PET, 15.2%). Twelve of the twenty-four detected adenoma patients underwent surgery, and in all cases, a pathological parathyroid adenoma was resected at the site detected by PET/CT. Two of the six patients without pathological choline uptake who received a parathyroidectomy revealed no evidence of parathyroid adenoma tissue in the histopathological evaluation. This retrospective study analyzes ^18^F-FCH PET/CT in a challenging patient cohort with pHPT and negative or equivocal conventional imaging results and supports the use of ^18^F-FCH for the diagnosis of hyperfunctional parathyroid tissue, especially in this patient setting, with a 100% true positive and true negative detection rate. Our study further demonstrates the importance of ^18^F-FCH PET/CT for successful surgical guidance.

## 1. Introduction

Primary hyperparathyroidism (pHPT) represents one of the most common endocrine disorders [1] with a prevalence of 3/1000 in the general population and a higher prevalence in postmenopausal females of up to 21/1000 [2,3]. With the exception of a few genetic cases of multiple endocrine neoplasms (MEN I and II) or a condition after neck irradiation, the etiological cause of pHPT usually remains unknown. In about 90% of cases, a solitary parathyroid adenoma is detected. In approximately 10% of patients, a multiglandular disease exists in the form of two or more adenomas or an adenoma combined with hyperplasia of another or more parathyroid glands, whereas parathyroid carcinoma is a rarity (<1%) [4].

The disease develops as a result of the autonomous production and secretion of the parathyroid hormone (PTH) from parathyroid glands. It is the most common cause of hypercalcemia and does not show specific symptoms for a long time; rather, it diffuses general symptoms, such as hypertension, nephrolithiasis, abdominal pain, and nausea, but ultimately patients often present decreased bone mineral density leading to fractures [5]. A diagnosis is primarily made as an incidental finding or as part of the workup for secondary osteoporosis. A diagnosis of pHPT is determined by laboratory results, including elevated serum calcium and PTH levels.

Once a diagnosis of symptomatic pHPT is established, the only definite therapy today is the removal of the affected parathyroid gland(s). If surgery is indicated, accurate preoperative localization of the hyperfunctioning gland(s) is a prerequisite for a minimally invasive parathyroidectomy, reducing operation time, complications, and hospitalization; improving postoperative recovery; and resulting in a better cosmetic outcome and greater patient satisfaction [6,7,8].

Various imaging methods have been used for the detection and localization of hyperfunctioning parathyroid glands in patients with pHPT, including morphological (ultrasounds, computed tomography, and magnetic resonance tomography), functional (scintigraphy), and hybrid techniques (PET/CT and PET/MRI) [9].

The currently most widespread preoperative imaging procedure is the combination of [^99m^Tc]Tc-MIBI scintigraphy and cervical ultrasonography (US), yielding a sensitivity of 84–88% [10,11,12], which is mainly contributed by [^99m^Tc]Tc-MIBI scintigraphy when combined with single-photon emission computed tomography/computed tomography (SPECT/CT). This implies that, in up to 20% of cases, a pathological parathyroid gland can be detected by neither ultrasound nor scintigraphy [13]. Of note, especially in cases of unsuccessful surgical exploration of the neck in pHPT (persistent pHPT) and in cases of recurrent pHPT, an exact localization diagnosis is urgently needed because surgical re-exploration is a difficult procedure with increased risks, such as recurrent paresis and hypoparathyroidism.

Further improvement of the detection accuracy of pathologic parathyroid glands with functional imaging is, therefore, desirable and essential for minimally invasive surgery or re-exploration. In an attempt to further increase the diagnostic performance of functional imaging for the localization of parathyroid adenoma, several PET tracers have been evaluated, and recently, ^18^F-Fluorocholine PET/CT, which was initially used to diagnose recurrent prostate cancer, was found to be able to identify pathological parathyroid glands [14,15,16,17]. First reported by Quack et al., choline PET/CT has since started to gain acceptance as a technique for the localization of parathyroid adenoma [18].

The uptake mechanism of ^18^F-Fluorocholine in parathyroid adenoma is thought to be driven by increased cell proliferation and metabolism, as well as the upregulation of choline kinase activity. As a phospholipid analogue, radio-labeled choline is integrated into newly synthesized membranes of proliferating cells, and its uptake is increased by the upregulation of choline kinase [19,20]. Interestingly, the upregulation of phospholipid-dependant choline kinase was shown to be related to PTH secretion in pHPT [21]. Based on this suggested mechanism, ^18^F-Fluorocholine has been used to study parathyroid adenoma.

In this study, we report the detection rate of ^18^F-Fluorocholine PET/CT in a challenging patient cohort with pHPT and inconclusive or negative findings in ultrasounds, [^99m^Tc]-MIBI scintigraphy, or previous resections.

## 2. Materials and Methods

### 2.1. Patient Selection

Patients who underwent ^18^F-Fluorocholine PET/CT for the evaluation of pHPT from November 2020 until February 2022 were retrospectively included in this study.

The inclusion criteria consisted of diagnosed pHPT by specialists in endocrinology according to current clinical guidelines. The patients were transferred to our clinic in case of contradictory diagnostic results or a lack of morphological evidence in standard imaging for parathyroid adenoma despite biochemical evidence of pHPT. Thus, this retrospectively collected cohort included patients with negative or equivocal results of ultrasonography and, in some cases, scintigraphy (MIBI) in whom it, therefore, was not possible to unambiguously locate the pathologic parathyroid gland(s). Patients with previous thyroidectomies or unsuccessful parathyroidectomies were classified as preoperated.

### 2.2. Informed Consent

All the patients signed a written informed consent to perform a PET/CT scan, and the local ethics committee approved this retrospective study (Number: 21-1203), including the retrospective analysis of the records. The study was performed in accordance with the Declaration of Helsinki.

### 2.3. Scan Acquisition and PET Image Interpretation

PET/CT images were acquired with a whole-body PET/CT scanner (Discovery PET/CT 690, GE Healthcare, Chicago, IL, USA) or a Siemens Biograph mCT flow (Siemens Healthineers, Erlangen, Germany). Images ranging from the temporomandibular joint to the diaphragm were acquired at a mean time of 56.8 ± 20 min after an injection of approximately 204.6 ± 36 MBq of ^18^F-Fluorocholine. The attenuation correction was based on CT. CT was performed as full-dose CT (automated dose modulation mean of 220 mAs, 120 kV, rotation time 0.5 s, 2 × 64 slights). A weight-adapted mean of 120 mL of iodine-containing contrast agent (Imeron 350 mg iodine/mL, Bracco Imaging Deutschland GmbH, Konstanz, Germany) was intravenously infused at a rate of 2.5 mL/s prior to the CT scan. The CT scan was initiated 50 s post-injection to depict the venous contrast phase. PET images were then obtained using a 3 min acquisition time per bed position. The images were sent to a dedicated workstation (Hermes Medical Solution), and the PET/CT images were analyzed by a resident physician, as well as two physicians who were board-certified in radiology and nuclear medicine with high levels of experience in the reading of PET/CT images. In the presence of significant, focal ^18^F-FCH uptake and corresponding nodular lesions in the CT image found in typical locations for orthotopic or heterotopic parathyroid tissues, the PET/CT images were rated as positive. If the tracer uptake was of low intensity but in typical cervical or mediastinal locations for parathyroid tissue without corresponding nodular lesions in the CT image, the studies were classified as equivocal. Lastly, PET/CT scans with physiological distributions of ^18^F-FCH without evidence of increased tracer uptake were classified as negative.

The acquired quantitative measurements of increased ^18^F-FCH and abnormal foci were the maximal standardized uptake value (SUV_max_), as well as the diameters of lesions, which were visible on the CT images. The mean standardized uptake value (SUV_mean_) of the thyroid gland was obtained using a 1 cm ROI positioned over the ipsilateral thyroid gland or, in cases of previous hemithyroidectomies, over the remaining thyroid tissue.

### 2.4. Performance Analysis

The ^18^F-FCH PET/CT imaging results were compared with the histopathological examination as the reference standard for the diagnosis of hyperfunctioning parathyroid tissue. The results of the ^18^F-FCH-PET/CT scans were classified as true positive if the regional tracer uptake correlated with the histological results of hyperfunctioning parathyroid tissue in the detected site on the PET/CT scan and classified as true negative for absent regional tracer uptake and histological findings of normal parathyroid tissue.

The SUV_max_ of the parathyroid adenoma was correlated to the CT diameter. The 95% confidence interval of the SUV_mean_ in the thyroid gland was calculated and used as a threshold for the definition of parathyroid adenoma tissue.

### 2.5. Statistical Analysis

The data are reported as means or medians ± standard deviation, ranges, or confidence interval as stated using SPSS (Version 27). GraphPad Prism (Version 8.4.3, GraphPad Software Inc., San Diego, CA, USA) was used for illustration of the results. A significance level of *p* < 0.05 was applied in all analyses.

## 3. Results

**Figure 1:**
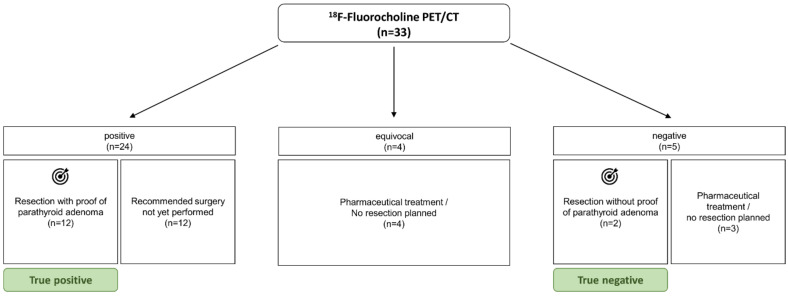
Patient selection flow chart.

A total of 33 patients underwent ^18^F-fluorocholine PET/CT from November 2020 until February 2022 and were retrospectively analyzed (Figure 1). The median age of the patients was 59.5 ± 13.3 years, and the female-to-male ratio of 22/11 represented the known prevalence. A high percentage of 11/33 (33.3%) patients had been operated on before. A pathological hyperfunctional parathyroid gland was detected in 24 cases (positive PET, 72.7%); 4 cases showed only weak, equivocal choline uptake (equivocal PET, 12.1%); and in 5 cases, no enhanced choline uptake was evident (negative PET, 15.2%) (Table 1).

### 3.1. Adenoma-Typical Choline Uptake Secures Resection of Parathyroid Adenoma

**Figure 2:**
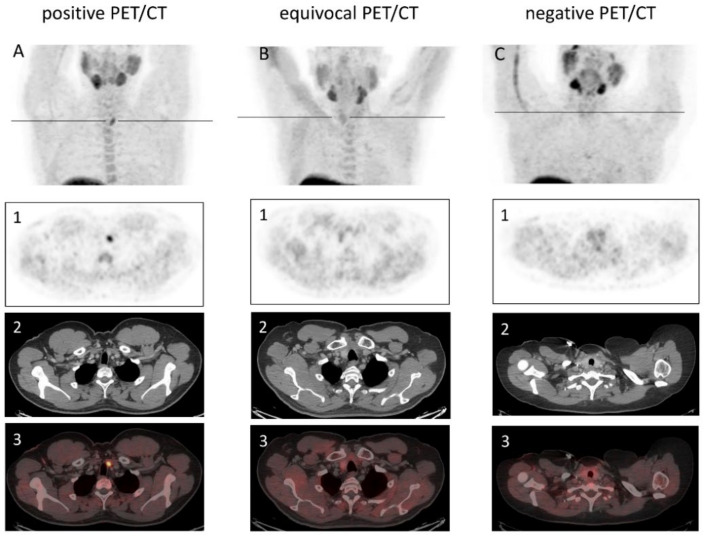
Patient examples of positive (**A**), equivocal (**B**), and negative (**C**) ^18^F-Fluorocholine PET/CT; PET (**1**), CT (**2**), and fused images of PET and CT (**3**). (**A**) a focal choline uptake correlating to a contrast enhanced lesion in the caudal left thyroid bed was detectable. (**B**) only weak and diffused choline uptake of the right thyroid tissue was visual. (**C**) no enhanced choline uptake was detectable.

In the 24 choline-avid lesions, the median SUV_max_ was 4.98 (range 2.43–9.99), and the median CT diameter amounted to 1.0 × 0.7 cm (range of 0.4 × 0.3 cm–1.9 × 2.1 cm; Figure 2). Of these 24 detected adenomas in choline PET/CT scans, only in 10 cases were questionable hypoechoic lesions discovered in ultrasonography, with a median diameter of 0.8 × 0.6 × 0.5 cm (range of 0.4 × 0.3 × 0.5 cm–2.0 × 1.1 × 2.4 cm). In the remaining 13 patients, no parathyroid gland was detectable on ultrasound, or no ultrasound was available (*n* = 2). Eight patients previously received [^99m^Tc]-MIBI scintigraphy with no sufficient evidence of prolonged uptake.

Twelve of the twenty-four detected adenoma patients underwent surgery, and in all cases, a pathological parathyroid adenoma was resected at the site detected by PET/CT. In the remaining 12/24 cases, surgery was recommended but not yet performed due to the COVID-19 pandemic and patient hesitation. One patient is now treated pharmacologically due to increased risk for surgery. Seven of the twenty-four patients had already been preoperated on prior to PET/CT. Two of these patients were successfully re-resected with histopathologically proven parathyroid adenoma tissue; the remaining five patients were suggested to receive surgery. One of the patients suffered from MEN-2A syndrome with two parathyroid adenomas in contralateral localizations, and one patient received a parathyroidectomy but did not show biochemical success, which was most likely due to a second parathyroid adenoma that was not depicted in the ^18^F-Fluorocholine PET/CT image.

### 3.2. Equivocal ^18^F-FCH PET/CTs Opt for Follow-Up and Pharmaceutical Treatments

The four patients with weak, equivocal choline uptake presented a median SUV_max_ of 3.76 (range of 3.30–4.33; Table 2). Two cases showed a doubtable intrathyroidal lesion on the CT image, but neither of the cases had a typical parathyroid correlate on a CT or ultrasound image. Choline uptake was partly evident intrathyroideal without possible discrimination of thyroid tissue and thyroid nodules. All four patients with only weak choline uptake had already undergone a parathyroidectomy or hemithyroidectomy, as well as [^99m^Tc]-MIBI scintigraphy, without any detection of prolonged uptake or suspected parathyroid adenoma prior to PET/CT. All the patients were receiving pharmacological treatment and regular follow-ups due to a lack of morphological findings in either ultrasound, [^99m^Tc]-MIBI scintigraphy, or PET/CT images.

### 3.3. Negative ^18^F-FCH PET/CT Studies Forecast Unsuccessful Surgery

Ultrasound images revealed no reliable morphological evidence of parathyroid adenomas in four patients. One patient presented a hypoechoic lesion without a corresponding choline uptake on the PET/CT scan. Two of the five patients without pathological choline uptake received parathyroidectomies afterwards, and histopathological evaluation revealed no evidence of parathyroid adenoma tissue in the PET negative operation field. Therefore, these patients were rated as true negative regarding the predictability of the operation. The remaining three patients were treated pharmacologically with regular follow-ups. Of note, one patient was already preoperated and received a re-resection after PET/CT scanning without evidence of parathyroid adenoma tissue.

### 3.4. Threshold-Based Delineation of Parathyroid Adenoma

For the delineation of parathyroid adenoma, the SUV_mean_ of the thyroid gland was measured, and the 95% confidence interval was calculated to differentiate between physiological thyroid tissue and pathological uptake in the parathyroid gland. The median SUV_mean_ of the thyroid gland in the overall group was 2.20 with a 95% confidence interval of 2.10–2.42 compared to the median SUV_max_ of parathyroid adenoma, with 4.36 and a 95% confidence interval of 4.12–5.59.

### 3.5. Larger CT Lesions Display Higher Choline Uptake

**Figure 3:**
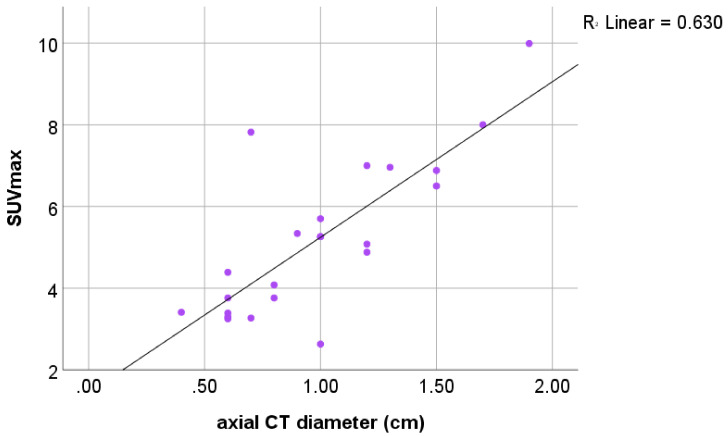
Significant positive correlation of the axial CT diameter (cm) with the SUV_max_ of parathyroid adenoma.

The axial CT diameter was measured in all the PET-positive cases. The axial CT diameter correlated significantly with the SUV_max_ in the parathyroid gland (*p* < 0.001; Figure 3). Of note, the smallest lesion, with a 0.4 cm diameter, presented an already intense uptake of SUV_max_ 3.41, which exceeded the calculated threshold of the thyroid gland of 2.42.

### 3.6. Laboratory Results

All patients presented pHPT, and in 28 patients, laboratory values were available with a mean total serum calcium of 2.78 ± 0.15 mmol/L (standard values: 2.05–2.65 mmol/L) and a mean parathyroid hormone (PTH) level of 127.76 ± 114.94 pg/mL (standard values: 15.0–65.0 pg/mL).

**Figure 4:**
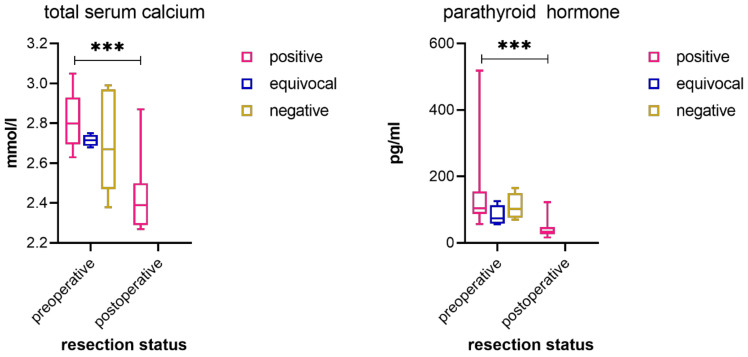
Laboratory results of the three groups of positive, equivocal, and negative PET/CT images. The group after successful resection showed a highly significant decrease in serum calcium and parathyroid hormone; *** = *p* < 0.001.

In 12 resected patients, postoperative total serum calcium and PTH levels were available, and in all despite one case, they dropped significantly to a physiological level: total serum calcium of 2.39 mmol/L (range of 2.27–2.62 mmol/L; *p* < 0.001) and PTH of 32.6 pg/mL (range of 16.8–53.6 pg/mL; *p* < 0.001; Figure 4). The one case showed a temporary decline in total serum calcium and PTH levels after resection but inclined again shortly afterwards, most likely due to a second, contralateral parathyroid adenoma that was not depicted by the PET/CT image.

In patients with positive PET/CT images, the values for serum calcium and PTH did not significantly differ compared to the overall group (serum calcium = 2.78 ± 0.15 mmol/L, *p* = 0.186; PTH = 127.76 ± 114.94 pg/mL, *p* = 0.335) or to the group with no evidence of adenoma in PET/CT images (serum calcium = 2.73 ± 0.22 mmol/L, *p* = 0.176; PTH = 105.52 ± 35.40 pg/mL, *p* = 0.531) (Figure 4).

## 4. Discussion

Conventional methods exhibit limited sensitivity for the localization of hyperfunctioning parathyroid adenoma, but prior knowledge is crucial to avoid re-operation and to reduce surgery time and morbidity. Conventional MRI and CT have low detection rates and are generally not recommended for the detection of parathyroid adenoma [22]. Four-dimensional CT provides an improved sensitivity of approximately 88% but results in a much higher radiation dose to the thyroid bed [23,24] compared to ^18^F-Fluorocholine PET/CT.

In a comparative study of surgical outcomes for patients with positive and negative localization diagnoses (MIBI, ultrasound, and CT), longer surgical times and significantly lower cure rates (87 vs. 96%) were found in patients with negative findings [25]. Of note, Karakas et al. reported that, for primary surgery (no preoperation on the thyroid or parathyroid gland) with negative standard diagnostics (ultrasound and MIBI), a bilateral exploration by an experienced endocrine surgeon led to success in about 98% of cases, i.e., the adenoma was found [26]. However, as mentioned above, minimally invasive parathyroidectomy is preferable to bilateral exploration of the neck, which has increased risk of surgery, longer operation time, complications, and hospitalization. In the case of a negative preoperative localization diagnosis (ultrasound or MIBI), the current German guidelines on surgery for pHPT recommend discussing with the patient individually the two options of bilateral surgical exploration with a slightly reduced healing rate or, alternatively, planning further localization diagnostics, e.g., choline PET/CT to allow a focused, minimally invasive parathyroidectomy with its advantages, if possible [27,28].

The reference standard for the imaging of the parathyroid gland is an ultrasound, which provides good yield when parathyroid adenomas are in typical localizations close to the thyroid gland (caudal and cranial poles), but parathyroid glands have multiple location variants for which the diagnostic rate of an ultrasound decreases. On the contrary, ultrasounds are very investigator-dependent and are often negative in cases of multiglandular disease or when adenomas are in atypical or ectopic localizations (e.g., mediastinum). ^18^F-FCH-PET/CT overcomes these difficulties, has a high detection rate, is independent of the location, and depends less on the experience of the reader.

Therefore, further improvement of preoperative imaging is of the utmost importance. In our study, we found a true positive rate of 100% in patients who were operated on after ^18^F-FCH PET/CT. Parathyroid adenoma was histopathologically proved at the site detected by PET/CT in all patients with positive PET/CT and subsequent resection, whereas no pathological parathyroid tissue or normalization of laboratory results was evident in patients with negative PET/CT, if resected. Of note, in one case, an intrathyroidal parathyroid adenoma was detected by PET/CT and successfully resected. We confirmed the superior diagnostic ability of this newer functional imaging technique, especially in patients with negative conventional imaging findings.

Four PET/CT scans were rated as equivocal. It is important to be aware that published acquisition protocols for ^18^F-FCH PET/CT vary considerably. The heterogeneous protocols include dynamic and static studies with single- or double-scan time points and acquisition at any time point between 2 and 120 min after injection. This can have an important impact on how images are interpreted and could bias their reported accuracy. In our study, the patients were scanned with a median time of 60 min after injecting the tracer. We cannot exclude that our equivocal cases could have been diagnosed more precisely at a different time point. Therefore, in further studies, we recommend a full dynamic analysis, which should clarify the best acquisition timing. Indeed, Prabhu et al. reported a higher uptake in parathyroid adenoma for scans obtained in the first 15 min after tracer injection compared with delayed static images 45–60 min after injection [29]. In contrast, Rep et al. demonstrated a marginally higher sensitivity and accuracy at 60 min in comparison to 5 min after injection [30]. Thus, they concluded that image acquisition was ideal at 60 min after fluorocholine administration to localize parathyroid glands prior operation.

A review by Evangelista et al. already reported that ^18^F-FCH PET/CT is more accurate than ^99m^Tc-MIBI scintigraphy in patients with negative or doubtful ultrasound findings [31]. PET has a limited spatial resolution and suffers from partial volume effects [32], especially in very small lesions, though in our cases, even very small lesions with an axial CT diameter <0.5 cm displayed pathological choline uptake and could be delineated, demonstrating a good detection rate even in small lesions.

Compared to our results, a small number of false negative or false positive findings have been reported in a couple of studies [16,18,33]. This may be due to the misinterpretation of thyroid anomalies or due to a higher uptake in thyroid nodules and goiter [31].

Goiter and thyroid adenoma can have pronounced, rather diffused choline uptake. To better differentiate the choline uptake in thyroid tissue compared to the parathyroid choline uptake, we measured the regional SUV_max_ of the parathyroid adenoma and compared it to the SUV_mean_ of the thyroid tissue. The SUV_max_ of the parathyroid adenoma was higher than the SUV_mean_ + 2 standard deviations of the thyroid tissue in all the cases, suggesting a >2 standard deviation threshold for the differentiation of parathyroid adenoma tissue. In line with our findings, Parvinian et al. previously described a 2.1 times higher uptake in the parathyroid gland compared to the adjacent thyroid tissue [34].

In our study, the group of patients with equivocal PET/CT results did received resection due to missing morphological imaging correlates. In the knowledge of the true negative results from the patient cohort without any correlates on PET/CT images, a watchful waiting strategy and pharmaceutical treatment might be valuable options, and the decision to perform a bilateral neck dissection should be discussed with the patients individually, as suggested by the German guidelines on surgery for pHPT [28]. Further studies have to evaluate if a follow-up with choline PET/CT after 6–12 months might increase the detection rate under the condition that the parathyroid adenoma might grow or increase its pathological function.

In patients with negative ^18^F-FCH PET/CTs, a pharmaceutical treatment with regular follow-ups might be superior to an explorative parathyroidectomy without a clear focus, since two of the patients received resections without preoperative focal detection on PET/CT images, and histopathologically, no parathyroid adenoma tissue was detected.

Especially in preoperated patients, an exploratory neck dissection should be avoided, and specific imaging with ^18^F-Fluorocholine PET/CT can increase the probability of localizing a hyperfunctional parathyroid adenoma for targeted parathyroidectomy.

An alternative PET/CT tracer for the imaging of parathyroid adenoma is ^11^C-Methionine, which has been used for the detection of parathyroid adenoma. However, several studies have shown the superiority of ^18^F-Fluorocholine with increased accuracy and sensitivity, which results in a higher detection rate [35,36]. In addition, the use of ^11^C-Methionine is restricted due to a very short half-life of only 20.38 min and required on-site production. A direct performance study between ^18^F-Fluorocholine and ^11^C-Methionine concluded a better performance of ^18^F-Fluorocholine, allowing the localization of parathyroid adenoma in 96% of patients [35].

A limitation of this study is that not all patients received resections after ^18^F-Fluorocholine PET/CT. On the other hand, the two cases in which this occurred could not be successfully cured and still suffered from pHPT after explorative resection. Based on these findings, the indication for explorative resection in patients with negative ^18^F-Fluorocholine PET/CT and negative ultrasonography should be carefully discussed, and further studies are required.

A limitation but, on the other hand, also a strength of this study is a patient cohort with 12 patients who had been operated on before. This is, of course, a preselected and biased patient cohort, but also the most challenging in clinical routine. We were able to detect an adenoma-like parathyroid uptake in 70% of all cases and in 79% of preoperated patients. A prospective study by Quack et al. reported comparable positive PET findings (76%) in their patient cohort with pHPT, which supports our findings [18].

The recent EANM Guidelines [37] and the ESE Educational Program of Parathyroid Disorders (PARAT) [38] have discussed and recommended ^18^F-Fluorocholine as an alternative first-line method whenever possible, especially in patients with negative or equivocal standard imaging findings, and our results supported this suggestion.

## Figures and Tables

**Table 1:** Patient characteristics.

	Overall Group	Positive PET	Equivocal PET	Negative PET
patients (*n*)	33	24	4	5
age (mean ± SD)	58.56 ± 14.10	60.24 ± 12.52	61.67 ± 6.68	53.97 ± 20.77
sex (female/male)	23/11	16/8	3/1	3/2
serum calcium preoperative (mmol/L)	2.78 ± 0.15	2.80 ± 0.15	2.72 ± 0.03	2.73 ± 0.22
serum calcium postoperative (mmol/L)	2.46 ± 0.20	2.44 ± 0.18	-	-
PTH preoperative (pg/mL)	127.76 ± 114.94	141.78 ± 134.24	81.95 ± 30.34	105.52 ± 35.40
PTH postoperative (pg/mL)	47.35 ± 34.17	41.33 ± 27.54	-	-
preoperated status (*n*)	12	7	4	1
resection (*n*)	14	12	0	2
histopathological evidence of parathyroid adenoma (*n*)	12	12	0	0

**Table 2:** Single-patient imaging parameters.

Patient #	US (0 = No Evidence; 1 = Evidence)	Preoperated (0 = No, 1 = Yes)	PET	SUV_max_	CT (0 = No Evidence; 1 = Evidence)	CT Diameter (Axial, cm)
1	1	0	positive	4.39	1	0.6 × 0.4
2	0	1	positive	3.76	1	0.6 × 0.4
3	0	0	negative	-	0	-
4	0	1	positive	4.88	1	1.2 × 0.7
5	0	0	positive	3.30	1	0.6 × 0.5
6	0	0	positive	8.00	1	1.7 × 1.0
7	1	0	positive	3.25	1	0.6 × 0.4
8	1	0	positive	9.99	1	1.9 × 2.1
9	1	1	positive	5.26	1	1.0 × 0.7
10	-	1	equivocal	3.76	1	0.8 × 1.0
11	0	0	negative	-	0	-
12	0	0	positive	3.27	1	0.7 × 0.5
13	0	1	positive	5.70	1	1.0 × 0.7
14	0	0	positive	3.39	1	0.6 × 0.4
15	1	0	positive	5.08	1	1.2 × 0.7
16	0	0	negative	-	0	-
17	1	0	positive	6.50	1	1.5 × 1.3
18	0	1	negative	-	0	-
19	0	1	equivocal	2.74	0	
20	-	0	positive	6.96	1	1.3 × 1.0
21	1	0	positive	2.43	0	-
22	1	0	positive	5.34	1	0.9 × 0.5
23	0	1	equivocal	3.30	0	-
24	0	0	positive	2.63	1	1.0 × 0.7
25	1	0	negative	-	0	-
26	1	0	positive	4.08	1	0.8 × 0.3
27	0	0	positive	3.41	1	0.4 × 0.5
28	0	1	positive	7.00	1	1.2 × 1.1
29	1	0	positive	6.88	1	1.5 × 1.0
30	0	1	positive	7.82	1	0.7 × 0.6
31	0	0	positive	3.19	0	-
32	0	1	equivocal	4.33	0	-
33	0	1	positive	5.26	1	1.0 × 0.7

## Data Availability

The data presented in this study are available on request from the corresponding author.
